# Spherical and Spindle-Like Abamectin-Loaded Nanoparticles by Flash Nanoprecipitation for Southern Root-Knot Nematode Control: Preparation and Characterization

**DOI:** 10.3390/nano8060449

**Published:** 2018-06-20

**Authors:** Zhinan Fu, Kai Chen, Li Li, Fang Zhao, Yan Wang, Mingwei Wang, Yue Shen, Haixin Cui, Dianhua Liu, Xuhong Guo

**Affiliations:** 1 State Key Laboratory of Chemical Engineering, East China University of Science and Technology, Shanghai 200237, China; fzn940223@163.com (Z.F.); chenkai@shzu.edu.cn (K.C.); lili76131@ecust.edu.cn (L.L.); fzhao1@ecust.edu.cn (F.Z.); mingweiwang@ecust.edu.cn (M.W.); dhliu@ecust.edu.cn (D.L.); 2 Engineering Research Center of Materials Chemical Engineering of Xinjiang Bingtuan, Shihezi University, Xinjiang 832000, China; 3 Environment and Sustainable Development in Agriculture, Chinese Academy of Agricultural Sciences, Beijing 100081, China; wangyan03@caas.cn

**Keywords:** *Meloidogyne incognita*, Abamectin, flash nanoprecipitation, amphiphilic block copolymers, spindle-like nanoparticles

## Abstract

Southern root-knot nematode (*Meloidogyne incognita*) is a biotrophic parasite, causing enormous loss in global crop production annually. Abamectin (Abm) is a biological and high-efficiency pesticide against *Meloidogyne incognita*. In this study, a powerful method, flash nanoprecipitation (FNP), was adopted to successfully produce Abm-loaded nanoparticle suspensions with high drug loading capacity (>40%) and encapsulation efficiency (>95%), where amphiphilic block copolymers (BCPs) poly(lactic-co-glycolic acid)-*b*-poly(ethylene glycol) (PLGA-*b*-PEG), poly(d,l-lactide)-*b*-poly(ethylene glycol) (PLA-*b*-PEG), or poly(caprolactone)-*b*-poly(ethylene glycol) (PCL-*b*-PEG) were used as the stabilizer to prevent the nanoparticles from aggregation. The effect of the drug-to-stabilizer feed ratio on the particle stability were investigated. Moreover, the effect of the BCP composition on the morphology of Abm-loaded nanoparticles for controlling *Meloidogyne incognita* were discussed. Notably, spindle-like nanoparticles were obtained with PCL-*b*-PEG as the stabilizer and found significantly more efficient (98.4% mortality at 1 ppm particle concentration) than spherical nanoparticles using PLGA-*b*-PEG or PLA-*b*-PEG as the stabilizer. This work provides a more rapid and powerful method to prepare stable Abm-loaded nanoparticles with tunable morphologies and improved effectiveness for controlling *Meloidogyne incognita*.

## 1. Introduction

Plant-parasitic nematodes are one of the major agricultural pests worldwide, which have caused in excess of $157 billion in global crops damage annually [1]. Nematodes show a wide variety of interactions with their hosts, among which parasitic worms never enter the host and simply migrate through the soil, using roots as an ephemeral food source [2]. In particular, the root-knot nematodes (*Meloidogyne incognita*) are biotrophic parasite that draw nutrition from the root of hosts with a rich food source for several weeks, causing premature death of the host and reducing crop yields [3]. Traditionally, plants are grown in the soil in which *Meloidogyne incognita* have been controlled through the use of synthetic nematicides, such as fumigant nematicides [4]. Nevertheless, the use of these types of pesticides is undesirable due to problems of residual toxicity, environmental pollution, and public health hazards, etc. Alternative non-fumigant nematicides have been used [5], but they are inefficient in the control of root-knot nematodes because of their rapid degradation by soil microorganisms after repetitive use [6]. One biological and high-efficiency non-fumigant nematicide, Abamectin (Abm), has been widely used to control *Meloidogyne incognita* since the early 1980s [7]. However, one basic and formidable problem of Abm is its low solubility in water. In many cases, it is applied in the form of oil in water emulsion [8], which shows limited steady shelf time and binds to organic contents in crops. Another drawback of Abm is its degradation by photo-oxidation [9]. As a result, in practical application, most of the applied Abm is lost because of degradation under UV irradiation. Hence, Abm’s dose is always increased to ensure efficacy, which results in increased costs and environmental pollution [10,11].

Nowadays, the rapid development of nanotechnology presents a new way to improve the performances of conventional pesticide formulations through the construction of nanotechnology-based agricultural systems such as the drug-carrier and a controllable drug targeting and releasing system [12,13,14,15,16,17,18,19,20]. Recently, there have been many types of nanomaterials for these nanotechnology-based systems, such as Ag-based nanocomposite structure for antibacterial [21], MoS_2_ nanosheets nanostructures for targeted chemotherapy [22], and amphiphilic block copolymers (BCPs) for stabilizing drugs. Generally, drug molecules can be encapsulated inside the formed nanoparticles by using biocompatible nanomaterial BCPs as the carrier and stabilizer to enhance the drug stability and bioavailability. The resulted drug-loaded nanoparticles are able to protect the Abm against degradation, control the release rate, and prolong the duration of efficacy of Abm pesticide formulations [23,24,25].

**Figure 1:**
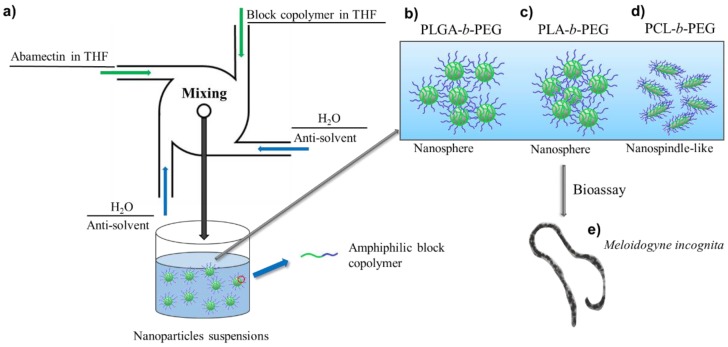
(**a**) Illustration of the preparation of Abamectin (Abm)-loaded nanoparticles by flash nanoprecipitation. (**b**–**d**) Morphology of Abm-loaded nanoparticles with different stabilizers: poly(lactic-co-glycolic acid)-*b*-poly(ethylene glycol) (PLGA-*b*-PEG) (**b**), poly(d,l-lactide)-*b*-poly(ethylene glycol) (PLA-*b*-PEG) (**c**), and poly(caprolactone)-*b*-poly(ethylene glycol) (PCL-*b*-PEG) (**d**). (**e**) Biological assay of Abm-loaded nanoparticles to *Meloidogyne incognita*. THF = tetrahydrofuran.

Flash nanoprecipitation (FNP) is a simple and generic method to rapidly construct nanosized drug-loaded particles by copolymer-directed assembly [26,27]. FNP involves rapid micromixing of organic solutions of the hydrophobic drug and BCP with water (anti-solvent) in a multi-inlet vortex mixer (MIVM) to create high supersaturations of the drug in milliseconds, and then rapidly form the hydrophobic core (drug) in the mixed solvent. These hydrophobic cores are subsequently stabilized and protected from aggregation by the BCP [28,29,30,31], as illustrated in Figure 1a. The FNP method has been demonstrated to be powerful for the preparation of drug-loaded nanoparticles with high drug-loading capacity, relatively narrow size distribution, and tunable nanometer particle size [32,33,34]. In FNP, the drug-loaded nanoparticles with spherical morphology using BCP as the stabilizer have been extensively studied both experimentally and theoretically. However, drug-loaded particles with non-spherical nanostructure obtained by FNP methods are seldom reported, although these particles have attracted increasing attention in biological field thanks to their unique features and properties, including longer blood circulation time, better motions under flow conditions, and stronger adhesiveness to the biological substrate [35,36]. Therefore, further study into FNP for a preparation method capable of controlling the morphology of drug-loaded nanoparticles is still needed.

In this study, Abm-loaded nanoparticles suspensions with excellent stability were generated via FNP. Three biocompatible BCPs, poly(lactic-co-glycolic acid)-*b*-poly(ethylene glycol) (PLGA-*b*-PEG), poly(d,l-lactide)-*b*-poly(ethylene glycol) (PLA-*b*-PEG), and poly(caprolactone)-*b*-poly(ethylene glycol) (PCL-*b*-PEG) with a molecular weight of 10k-*b*-5k were chosen as the stabilizers. The Abm-to-stabilizer feed ratio was optimized, and Abamectin loading capacity and encapsulation efficiency were evaluated. Spherical and spindle-like morphologies were observed for the obtained Abm-loaded nanoparticles (NPs) by FNP (FNP-NPs). The toxicity of Abm-loaded nanoparticles with these two morphologies on *Meloidogyne incognita* was investigated.

## 2. Materials and Methods

### 2.1. Materials

Abamectin (95.5%) was supplied by Hebei Veyong Bio-Chemical Co., Ltd. (Shijiazhuang, China). Poly(lactic-co-glycolic acid)-*b*-poly(ethylene glycol) (PLGA-*b*-PEG, 10k-*b*-5k) and poly(d,l-lactide)-*b*-poly(ethylene glycol) (PLA-*b*-PEG, 10k-*b*-5k) were purchased from Jinan Daigang Biomaterial Co., Ltd. (Jinan, China); ε-Caprolactone (ε-CL), stannous octoate, anhydrous toluene, and monomethoxy poly(ethylene glycol) (MW = 5000) were purchased from Sigma-Aldrich; diethyl ether was purchased from Shanghai Tianlian Fine Chemical Co., Ltd. (Shanghai, China), and tetrahydrofuran (THF) was purchased from Beijing Chemical Reagents Company (Beijing, China). Poly(caprolactone)-*b*-poly(ethylene glycol) (PCL-*b*-PEG, 10k-*b*-5k) was synthesized according to a previously reported method [32].

A population of root-knot nematodes (*Meloidogyne incognita*) was originally obtained from a greenhouse in the Beijing Academy of Agriculture and Forestry, Beijing, China.

### 2.2. Preparation of Abm-Loaded Nanoparticles

Abm-loaded nanoparticles were prepared by FNP method using a four-stream MIVM (Figure 1a). The amphiphilic block copolymer (PLGA-*b*-PEG, PLA-*b*-PEG, or PCL-*b*-PEG; 10k-*b*-5k) and Abamectin were dissolved in THF. Different drug-to-stabilizer feed ratios (weight) were used: 1:10, 2.5:10, 7.5:10, and 10:10. Solutions of Abm and BCP in THF (streams 1 and 2, 25 °C) were fed at the same flow rate (12 mL/min) along with two other deionized water streams (streams 3 and 4, 25 °C) both at a flow rate of 24 mL/min into the MIVM. The corresponding Reynolds number (*Re*) was calculated to be 5962, which is within the turbulent flow region with better mixing. The concentrations of Abm were 1, 2.5, 7.5, or 10 mg/mL, and the BCP concentration was fixed at 10 mg/mL. It is important to note that the concentrations of Abm and BCP in final nanoparticle solution was decreased due to the merging of the four streams. In addition, the morphology of FNP-NPs was strongly influenced by the glass transition temperature (*T*_g_) of the BCP used, which affects the assembly of BCP on the hydrophobic core (drug) during FNP, and a low *T*_g_ could result in non-spherical nanoparticles under the millisecond mixing conditions of FNP [33]. In order to investigate the potential of FNP to form Abm-loaded nanoparticles with non-spherical morphologies, we used different BCPs with an appropriate hydrophilic to hydrophobic block molecular weight ratio.

### 2.3. Characterization

Particle size and size distribution were measured by dynamic light scattering (DLS) with a ZetasizerNano ZS90 (Malvern Instruments, ‎Malvern, UK). The light intensity correlation function was collected at 25 °C with a scattering angle of 90°. The values obtained were averaged from three duplicates. Nanoparticle morphology was observed by transmission electron microscopy (TEM) (Hitachi HT7700, Hitachi Ltd., Chiyoda-ku, Japan) with an acceleration voltage of 80 kV. The samples were prepared by dripping the fresh solution onto carbon-coated copper grids and then dried overnight at room temperature. The amount of drug loaded in nanoparticles was determined by drug absorption at 245 nm using high performance liquid chromatography (HPLC) (Aglient 1260, ‎Santa Clara, CA,‎ USA) with a C18 column (5 µm, 4.6 mm × 150 mm, Aglient, Santa Clara, CA,‎ USA) and a 245-nm UV detector.

### 2.4. Drug Loading Capacity (DLC) and Encapsulation Efficiency (EE)

To determine the DLC and EE of Abm in Abm-loaded nanoparticles, the nanoparticle solutions with a known volume (~5 mL) were dialyzed for 24 h using a dialysis bag (molecular weight cut off: 8-14 kDa), and the Milli-Q water was changed six times to remove the organic solvent down to a barely detectable level (i.e., <0.008 *v*/*v* %). For each change of Milli-Q water, the volume of water in the dialysis bag was slightly increased. After removal of the free Abm and residual organic solvent by dialysis, Abm concentration was examined by HPLC at 245 nm. The solution was diluted by methanol and filtered through a 0.2-µm filter prior to HPLC analysis. Then, the DLC and EE of Abm-loaded nanoparticles were calculated according to the following equations:

$$\mathrm{DLC}(\%)=\frac{\mathrm{Total}\;\mathrm{mass}\;\mathrm{of}\;\mathrm{loaded}\;\mathrm{Abamectin}}{\mathrm{Total}\;\mathrm{mass}\;\mathrm{of}\;\mathrm{nanoparticles}}\times 100$$

$$\mathrm{EE}(\%)=\frac{\mathrm{Total}\;\mathrm{amount}\;\mathrm{of}\;\mathrm{loaded}\;\mathrm{Abamectin}}{\mathrm{Total}\;\mathrm{amount}\;\mathrm{of}\;\mathrm{Abamectin}\;\mathrm{added}}\times 100$$

The samples after dialysis were packed in glass tubes which were then stored at 0 °C for 7 days or 54 °C for 14 days, and the changes in DLC in the Abm-loaded nanoparticles were studied.

### 2.5. Biological Assay

All juveniles were hatched in a sieve (mesh number 500) at 28 °C for 3 days, and then juveniles were collected and used in the following experiments.

The nematicidal efficacy of Abamectin against *Meloidogyne incognita* was determined in aqueous tests. Abamectin nanoparticle suspensions formulations (1, 0.5, 0.25, 0.125, and 0.0625 mg/L) were prepared by diluting the nanoparticle solution directly obtained from FNP with sterile water, and pure sterile water was used as the control. Then, 0.5 mL of the resulting solution and 0.5 mL of root-knot nematodes juveniles (containing an average of 50 juveniles) were added to each well of a 24-well plate, respectively. Well plates were wrapped and kept at 28 °C. After 24 h, the relative percentages of the motile and immotile juveniles were evaluated by observation under a microscope (Olympus, Tokyo, Japan) three times. Each experiment had four duplicates and the final results were obtained by averaging.

## 3. Results and Discussion

### 3.1. Effect of BCP on Size and Morphology of Abm-Loaded Particles

An important finding associated with BCP is that they have the potential to control the particle morphology and size when they assemble on the particle core (drug). Most of the Abm-loaded particles reported have spherical morphology, which is most likely to occur [23,37,38]. For many applications, however, non-spherical structures are desired, making it important to control the particle morphology.

**Figure 2:**
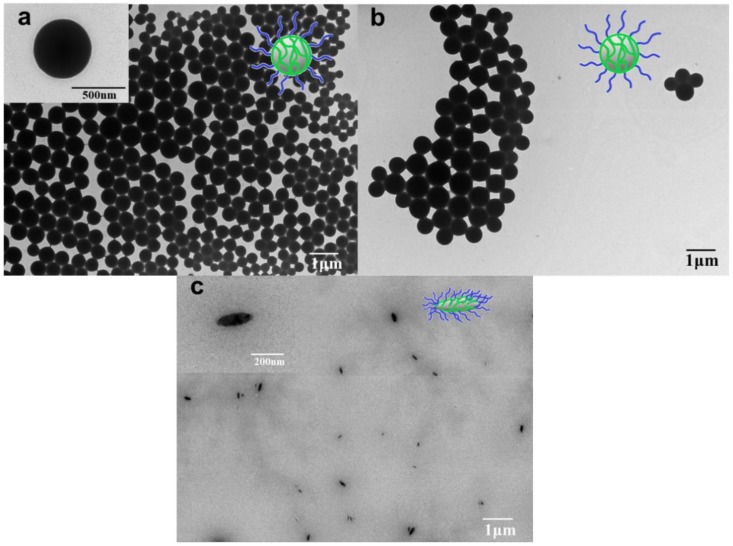
TEM photographs of Abm-loaded nanoparticles prepared using (**a**) PLGA-*b*-PEG, (**b**) PLA-*b*-PEG, and (**c**) PCL-*b*-PEG as the stabilizer, respectively. The insets are the corresponding schematic diagrams.

Focusing on preparing non-spherical particles, we found that the particle morphology can be controlled by using different BCPs as the stabilizer in FNP. Figure 2a,b show the typical TEM images of the prepared Abm-loaded nanoparticles using PLGA-*b*-PEG and PLA-*b*-PEG as stabilizers by FNP, respectively. The morphology of these particles is perfectly spherical and the particle size distribution is narrow, which is favorable for improving the dispersion, adhesion, and permeability of pesticides to target crops as a pesticide carrier [39]. Interestingly, as the BCP was changed to PCL-*b*-PEG, spindle-like particles were obtained (Figure 2c). The resulting nanoparticles have a long axis length ranging in 190–210 nm and a short axis length ranging in 70–80 nm instead of a normal spherical structure. Our observation of spindle-like Abm-loaded particles is an important finding because it is the first demonstration that the non-spherical drug-loaded particles are produced through FNP method. This finding also confirms that the spindle-like shape of PCL-*b*-PEG stabilized Abm-loaded nanoparticles due to the lower *T*_g_ of the hydrophobic PCL block, which causes the larger degree of stretching of the poly(caprolactone) (PCL) block. Meanwhile, the degree of stretching of the PCL block is too high to support the spherical morphology [40], leading to spindle-like morphology of the FNP-NPs. In addition, it is noteworthy that the edges of the nanoparticles were not smooth. This may be due to the presence of poly(ethylene glycol) (PEG) blocks which could affect the folding of the PCL chain [41]. Changes in drug-loaded particle shape could significantly change the drug release behavior, as suggested in the literature [42]. Thus, the relevance of spherical and non-spherical morphologies of Abm-loaded FNP-NPs and their nematicidal activity will be discussed in detail in a later section.

**Figure 3:**
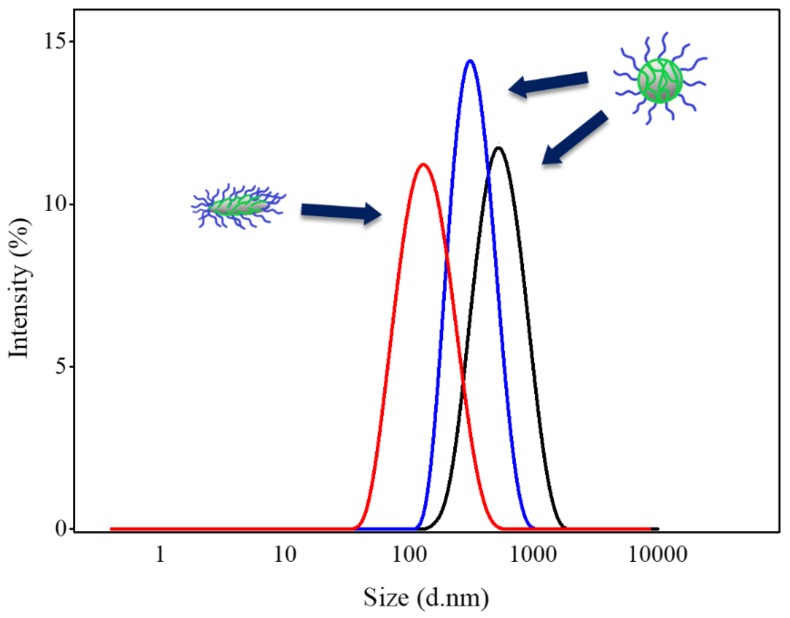
Particle size and size distribution of Abm-loaded nanoparticles prepared using PLGA-*b*-PEG (**black**), PLA-*b*-PEG (**blue**), and PCL-*b*-PEG (**red**) as the stabilizer, respectively. The mean diameters derived from the Gaussian fits (solid lines) are 414, 314, and 72 nm, respectively.

Figure 3 displays the average size (diameter) and size distribution of the prepared Abm-loaded particles when different BCPs were used as the stabilizer. It can be seen that the three kinds of FNP-NPs all had relatively narrow size distribution, validating the superiority of FNP. Meanwhile, it can be seen that PCL-*b*-PEG stabilized nanoparticles have smaller average sizes (72 nm) than those stabilized by PLGA-*b*-PEG or PLA-*b*-PEG (with an average size 414 or 314 nm). The most likely reason is that PCL, the hydrophobic block, has a much lower *T*_g_ (about −60 °C), making the BCP chain softer. Consequently, the drug is wrapped very rapidly, hindering the further growth of the drug nuclei and resulting in a smaller particle size. In contrast, PLGA-*b*-PEG and PLA-*b*-PEG have hydrophobic blocks with much higher *T*_g_ values (about 39 °C and 34 °C for poly(lactic-co-glycolic acid) (PLGA) and poly(d,l-lactide) (PLA), respectively) [43,44]. Thus the chains of these two BCPs are not flexible enough to encapsulate the drug nuclei in time before it grows larger, leading to much larger particle sizes.

Meanwhile, as suggested in the literature, smaller sizes (with large surface-volume ratios) could provide better performance, such as easy attachment and unique optical properties [45]. Therefore, the small spindle-like Abm-loaded nanoparticles obtained in our work could have higher potential in the control of *Meloidogyne incognita* (which will be validated in a later section).

### 3.2. Effect of Abm-to-Stabilizer Feed Ratio on Particle Stability

**Figure 4:**
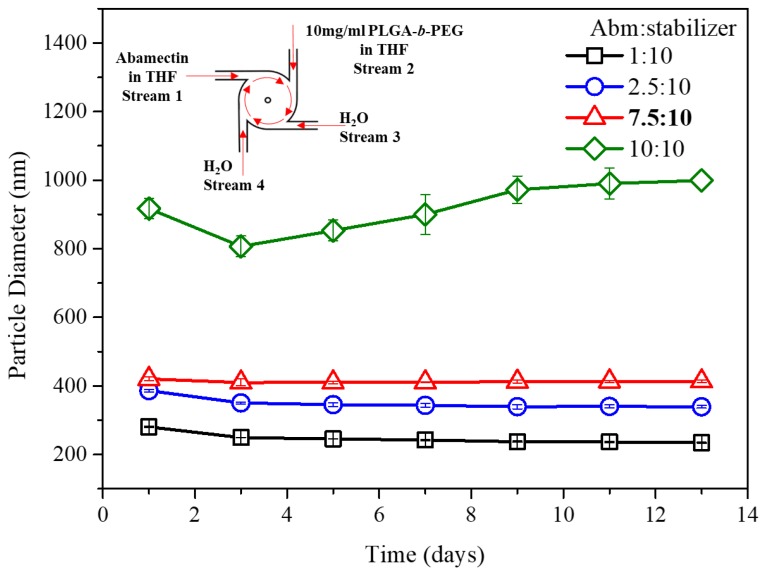
Effect of various Abm-to-stabilizer ratios on particle stability for flash nanoprecipitation-nanoparticles (FNP-NPs) prepared with 10 mg/mL PLGA-b-PEG as the stabilizer. Stream 1 was 1, 2.5, 7.5, or 10 mg/mL of Abamectin dissolved in THF. Stream 2 was 10 mg/mL of PLGA-*b*-PEG dissolved in THF. The other two streams were both water.

The dependence of particle size on feed ratio (weight) of Abm-to-stabilizer was exploited to optimize the particle stability which could be indicated by the change of particle size over time (the change of drug loading capacity along with time will be discussed in a later section). Abm-loaded nanoparticles prepared with different Abm-to-stabilizer feed ratios and with PLGA-*b*-PEG, PLA-*b*-PEG, or PCL-*b*-PEG as the stabilizer were stored at room temperature, and their particle size was monitored at defined time intervals. As shown in Figure 4 (with PLGA-*b*-PEG as the stabilizer), a feature that we saw for all the nanoparticles in the first three days was the “anti-Ostwald” phenomenon, which may be related to polar hydroxyl of Abm in nanoparticles. This polar moiety slowly rearranges towards the particle interface over time to minimize the energy of the system by increasing the surface to volume area, resulting in a decrease in particle size [46].

After the first three days, a normal feature that we saw for nanoparticles with a feed ratio (weight) of 10:10 (Abm/PLGA-*b*-PEG) was “Ostwald ripening” [46,47]. This is because the amount of stabilizer was not enough to encapsulate the drug nuclei in time to prevent their further growth with the high drug-to-stabilizer feed ratio 10:10, leading to the relatively wide size distribution of the initially obtained particles (polydispersity index (PDI) = 0.53, Table 1). Consequently, small nanoparticles would continuously dissolve to precipitate again on the surface of larger nanoparticles, and the average particle size increased gradually as shown in Figure 4. In contrast, particle stability was considerably enhanced when the Abm-to-stabilizer ratio was reduced to 7.5:10, 2.5:10, or 1:10, and the particle size remained essentially unchanged 10 days later. In addition, Abm loading increased with an increase in Abm-to-stabilizer feed ratio. This means the two lower Abm-to-stabilizer ratios (2.5:10 and 1:10) were inefficient at encapsulating Abm in nanoparticles. Hence, exhibiting remarkable stability and excellent drug loading capacity, the nanoparticles prepared with an Abm-to-stabilizer feed ratio of 7.5:10 (weight) were taken as the optimized formulation.

### 3.3. The Amount of Abamectin Encapsulated in FNP-NPs

The solubility of Abamectin in water is extremely low, which results in precipitation of Abamectin. During FNP, almost all of the Abamectin (>99.9%) could be encapsulated and stabilized by the stabilizer, which means Abamectin molecules could be wrapped in nanoparticles efficiently with nearly no loss during dialysis [48].

Thus, high drug loading capacity is one of the advantages of FNP. The amounts of Abamectin in the FNP-NPs (DLC and EE) using different stabilizers, are summarized in Table 2. The concentration of Abamectin in the final nanoparticle solution reached around 0.8 mg/mL after dialysis. The encapsulation efficiency was all higher than 95% using the three different stabilizers (Table 2). The slight loss may be caused by imperfect operation in the mixer MIVM, in which fluids may have hold-up volume. The values of DLC of different particles stabilized by PLGA-*b*-PEG, PLA-*b*-PEG, and PCL-*b*-PEG were determined to be 41.46%, 40.97%, and 40.76%, respectively. Overall, high drug loading capacity and encapsulation efficiency were achieved by the FNP method for all stabilizers at room temperature.

### 3.4. Effect of Temperature on Particle Stability

**Figure 5:**
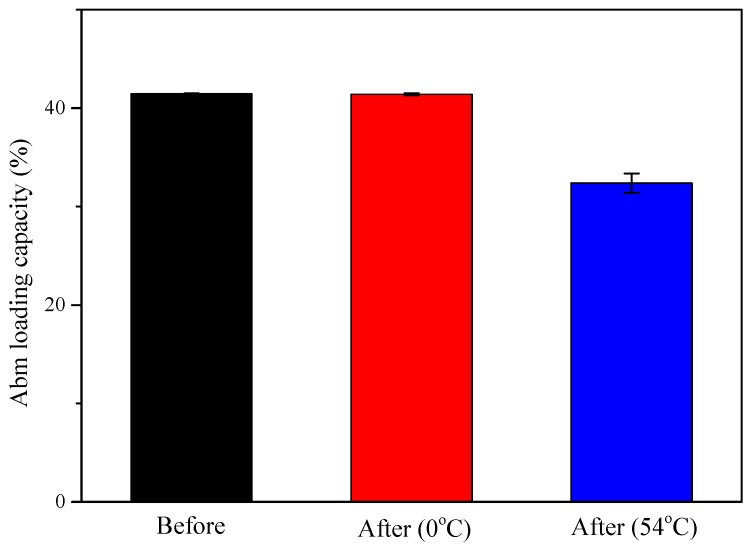
Abm loading capacity of the nanoparticles prepared using PLGA-b-PEG as the stabilizer before and after storage at 0 °C for 7 days and 54 °C for 14 days.

The storage stability of Abm-loaded nanoparticles was studied by measuring Abm loading capacity at two different temperatures (0 °C and 54 °C). The results are shown in Figure 5 (with PLGA-*b*-PEG as the stabilizer). Pleasingly, the Abm-loaded nanoparticles remained stable with no major changes in the loading capacity during storage at 0 °C, although the loss of Abm (9.09%) was found after storage for 14 days at 54 °C. The reason is that Abamectin degrades much faster at higher temperatures. These results show that the Abm-loaded nanoparticles can be kept in a very stable state during long-time storage at relatively low temperatures.

### 3.5. Toxicity of Abm-Loaded Particles to Meloidogyne incognita

**Figure 6:**
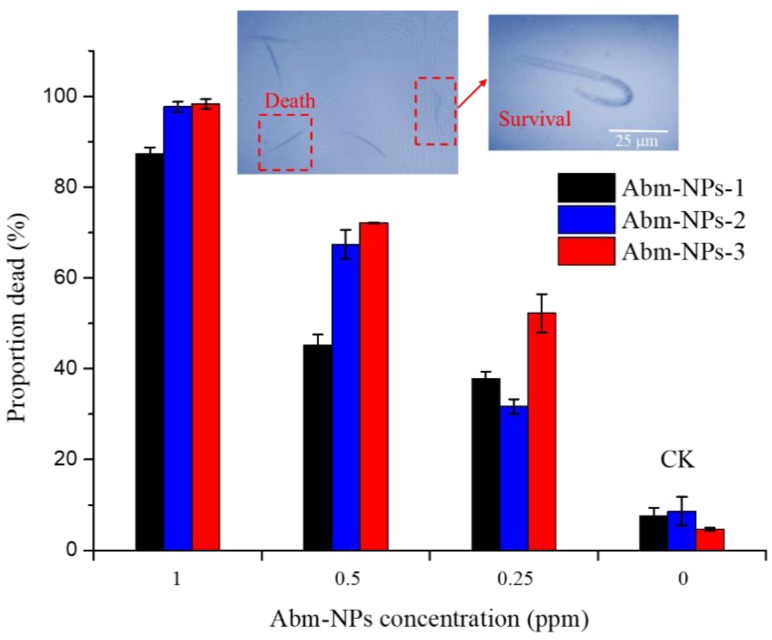
Mortality of *Meloidogyne incognita* as a function of concentration of Abm-loaded nanoparticles prepared using different block copolymers (BCPs) as the stabilizer.

As shown in Figure 6, all Abm-loaded nanoparticles still cause high mortality (87.2%, 97.7%, and 98.4% with PLGA-*b*-PEG, PLA-*b*-PEG, and PCL-*b*-PEG as the stabilizer, respectively) of *Meloidogyne incognita* even after being diluted 800 times (800 ppm to 1 ppm). The lethal concentrations required to kill 50% (LC_50_) (after 24 h) for *Meloidogyne incognita* were calculated and are shown in Table 3. The values of LC_50_ were 0.42, 0.37, and 0.28 ppm with PLGA-*b*-PEG, PLA-*b*-PEG, and PCL*-b*-PEG as the stabilizer, respectively (Table 3). The mean diameters of the three nanoparticles are 414, 314, and 72 nm, respectively (Figure 3), and they have the same Abm concentrations (0.8 mg/mL), but different LC_50_ values. One possible reason is that nanoparticles formed from BCPs containing a hydrophobic block (PLGA or PLA) with a high *T*_g_ tend to disassemble more slowly than those formed from BCPs with a low *T*_g_ hydrophobic block (PCL) [49]. Another possible reason is that the spindle-like nanoparticles obtained from PCL*-b*-PEG with smaller sizes have better adhesion and permeability.

Overall, the lethal concentration assays indicate that spindle-like Abm-loaded nanoparticles prepared using PCL-*b*-PEG as the stabilizer by FNP have higher nematicidal efficiency than spherical Abm-loaded nanoparticles using PLGA-*b*-PEG or PLA-*b*-PEG as the stabilizer. In addition, Figure 6 shows that the Abm-loaded nanoparticles caused irreversible paralysis in *Meloidogyne incognita*, and the nematode mortality increased with the increasing concentration of Abm-loaded nanoparticles. Thus, the nematode control effectiveness can be improved by using increased concentration of Abm-loaded nanoparticles [50].

## Figures and Tables

**Table 1:** Nanoparticle average size and size distribution with different Abm-to-stabilizer ratios (using PLGA-*b*-PEG as the stabilizer and the stabilizer concentration in THF before mixing was 10 mg/mL). PDI = polydispersity index.

Ratio of Abamectin to Stabilizer	Particle Diameter (nm)	PDI
1:10	252 ± 1	0.25 ± 0.01
2.5:10	355 ± 3	0.14 ± 0.04
7.5:10	414 ± 5	0.19 ± 0.07
10:10	898 ± 30	0.53 ± 0.20

**Table 2:** The amount of Abamectin encapsulated in Abm-NPs (Abm-to-stabilizer feed ratio 7.5:10). DLC = Drug Loading Capacity; EE = Encapsulation Efficiency.

	Stabilizer	DLC (%)	EE (%)
Abm-NPs-1	PLGA-*b*-PEG	41.46 ± 0.05	96.74 ± 0.12
Abm-NPs-2	PLA-*b*-PEG	40.97 ± 0.05	95.60 ± 0.11
Abm-NPs-3	PCL-*b*-PEG	40.76 ± 0.03	95.10 ± 0.07

**Table 3:** Bioassay results of the three Abamectin formulations. LC_50_ = lethal concentrations required to kill 50%. CL = confidence limit. DF = degree freedom. *P* = probability. *n* = number.

Population	Insecticide	Stabilizer	LC_50_ (95% CL *^a^*) (ppm)	Fit of Probit Line				
				Slope ± SE	*χ* ^2^	DF	*P*	*n ^b^*
Lab	Abm-NPs-1	PLGA-*b*-PEG	0.42 (0.32–0.62)	5.76 ± 0.33	5.95	3	0.11	252
Lab	Abm-NPs-2	PLA-*b*-PEG	0.37 (0.32–0.48)	6.72 ± 0.59	0.79	3	0.84	320
Lab	Abm-NPs-3	PCL-*b*-PEG	0.28 (0.23–0.33)	6.57 ± 0.33	3.45	3	0.32	352

*^a^* 95% confidence limit. *^b^* Number of larvae used in the bioassay.
